# Cytosinium–hydrogen maleate–cytosine (1/1/1)

**DOI:** 10.1107/S1600536809046571

**Published:** 2009-11-11

**Authors:** Nourredine Benali-Cherif, Wahiba Falek, Amani Direm

**Affiliations:** a Laboratoire des Structures, Propriétés et Interactions Inter-Atomiques, Centre Universitaire Abbes Laghrour, Khenchela 40000, Algeria; b Département de Chimie, Université Elhadj Lakhdar, Batna 05000, Algeria

## Abstract

The title organic salt, C_4_H_6_N_3_O^+^·C_4_H_3_O_4_^−^·C_4_H_5_N_3_O, was synthesized from cytosine base and maleic acid. An intra­molecular O—H⋯O hydrogen bond occurs in the hydrogen maleate anion. The crystal packing is stabilized by inter­molecular N—H⋯O, N—H⋯N and C—H⋯O hydrogen bonds, giving rise to a nearly planar two-dimensional network parallel to (101).

## Related literature

For background to cytosine, see: Devlin (1986 ▶); Johnson & Coghill (1925 ▶); Mahan *et al.* (2004 ▶). For the structure of cytosine, see: Barker & Marsh (1964 ▶) and for that of cytosine monohydrate, see: Jeffrey & Kinoshita (1963 ▶); Swamy *et al.* (2001 ▶). For the stuctures of inorganic cytosinium salts, see: Mandel (1977 ▶); Cherouana *et al.* (2003 ▶); Jaskólski (1989 ▶); Bagieu-Beucher (1990 ▶) and for those of cytosinium salts of organic acids, see: Gdaniec *et al.* (1989 ▶); Smith *et al.* (2005 ▶); Balasubramanian *et al.* (1996 ▶). For the hydrogen maleate anion, see: Madsen & Larsen (1998 ▶). For hydrogen-bond motifs, see: Bernstein *et al.* (1995 ▶).
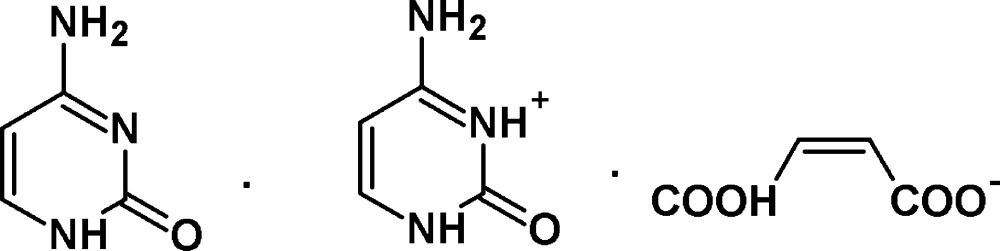


## Experimental

### 

#### Crystal data

C_4_H_6_N_3_O^+^·C_4_H_3_O_4_^−^·C_4_H_5_N_3_O
*M* *_r_* = 338.29
Monoclinic, 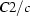

*a* = 27.3226 (5) Å
*b* = 7.3618 (2) Å
*c* = 14.6742 (4) Å
β = 93.905 (1)°
*V* = 2944.77 (13) Å^3^
*Z* = 8
Mo *K*α radiation
μ = 0.13 mm^−1^
*T* = 298 K
0.3 × 0.15 × 0.1 mm

#### Data collection

Nonius KappaCCD diffractometer
Absorption correction: none
3490 measured reflections
3485 independent reflections
2603 reflections with *I* > 2σ(*I*)
*R*_int_ = 0.043

#### Refinement

*R*[*F* ^2^ > 2σ(*F* ^2^)] = 0.048
*wR*(*F* ^2^) = 0.136
*S* = 1.07
3485 reflections
202 parameters
H atoms treated by a mixture of independent and constrained refinement
Δρ_max_ = 0.36 e Å^−3^
Δρ_min_ = −0.23 e Å^−3^

### 

Data collection: *KappaCCD Server Software* (Nonius, 1998 ▶); cell refinement: *DENZO* and *SCALEPACK* (Otwinowski & Minor, 1997 ▶); data reduction: *DENZO* and *SCALEPACK*; program(s) used to solve structure: *SIR2004* (Burla *et al.*, 2005 ▶); program(s) used to refine structure: *SHELXL97* (Sheldrick, 2008 ▶); molecular graphics: *ORTEP-3* (Farrugia, 1997 ▶) and *PLATON* (Spek, 2009 ▶); software used to prepare material for publication: *WinGX* (Farrugia, 1999 ▶).

## Supplementary Material

Crystal structure: contains datablocks global, I. DOI: 10.1107/S1600536809046571/dn2509sup1.cif

Structure factors: contains datablocks I. DOI: 10.1107/S1600536809046571/dn2509Isup2.hkl

Additional supplementary materials:  crystallographic information; 3D view; checkCIF report

## Figures and Tables

**Table 1:** Hydrogen-bond geometry (Å, °)

*D*—H⋯*A*	*D*—H	H⋯*A*	*D*⋯*A*	*D*—H⋯*A*
N1*A*—H1*A*⋯O4	0.86	1.89	2.7426 (19)	174
N1*B*—H1*B*⋯O2^i^	0.86	1.91	2.7701 (19)	174
N8*A*—H8*A*1⋯O7*B*	0.86	2.00	2.8582 (19)	178
N8*A*—H8*A*2⋯O7*A* ^ii^	0.86	2.04	2.8329 (19)	153
N3*B*—H3*B*⋯N3*A*	0.86	1.98	2.8370 (19)	176
N8*B*—H8*B*1⋯O7*A*	0.86	1.99	2.8458 (19)	173
N8*B*—H8*B*2⋯O7*B* ^iii^	0.86	2.06	2.8491 (18)	153
O3—H3⋯O1	1.17 (2)	1.25 (2)	2.4167 (16)	173 (2)
C6*B*—H6*B*⋯O1^i^	0.93	2.50	3.186 (2)	131
C5*B*—H5*B*⋯O2^iv^	0.93	2.42	3.330 (2)	165
C5*A*—H5*A*⋯O4^ii^	0.93	2.37	3.296 (2)	175

Symmetry codes: (i) 
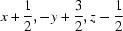
; (ii) 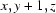; (iii) 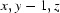; (iv) 
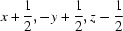
.

**Fig. 1.**
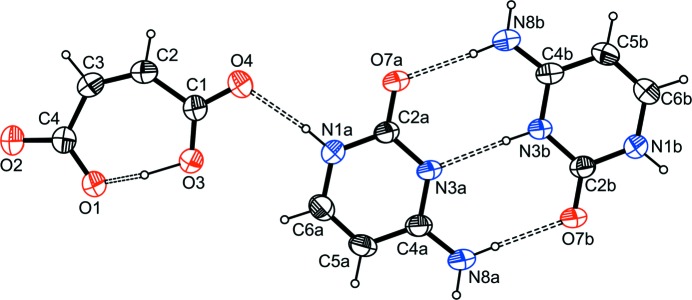
ORTEP view of the asymmetric unit of (I) with the atom labelling scheme. Displacement ellipsoids are drawn at the 50% probability level. H atoms are represented as small spheres of arbitrary radii. Hydrogen bonds are shown as dashed lines.

**Fig. 2.**
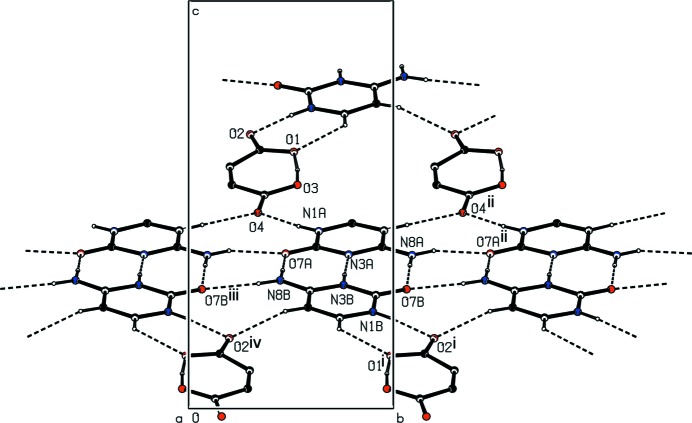
Partial packing view showing the formation of the two dimensionnal network through N-H···O, N-H···N and C-H···O hydrogen bonds. H atoms not involved in hydrogen bondings have been omitted for clarity. Hydrogen bonds are shown as dashed lines. [Symmetry codes: (i) x+1/2, -y+3/2, z-1/2; (ii) x, y+1, z; (iii) x, y-1, z; (iv) x+1/2, -y+1/2, z-1/2]

## supplementary crystallographic information

### Comment

The pyrimidine base, Cytosine, leads to the nucleoside cytidine and its corresponding nucleotide: cytidine 5'-monophosphate. It may be found in very small quantities as a post-modified form, 5-methylcytosine, in certain nucleic acids (Devlin, 1986) such as in tuberculinic acid (Johnson & Coghill, 1925). More recently, 5-fluoro-cytosine (5-FC) has been used as a prodrug in suicide gene therapy of cancer with the crystal structure of bacterial cytosine deaminase (bCD) (Mahan *et al.*, 2004).

The crystal structures of cytosine (Barker & Marsh, 1964) and cytosine monohydrate (Jeffrey & Kinoshita, 1963) were determined many years ago. (Swamy *et al.*, 2001)]. Many inorganic cytosinium salts have been previously synthesized: chloride (Mandel, 1977), nitrate (Cherouana *et al.*, 2003) and dihydrogenphosphate (Jaskólski, 1989; Bagieu-Beucher, 1990). Cytosinium salts of organic acids are also common, the structures of a number of these including trichloroacetate (Gdaniec *et al.*, 1989), Cytosinium 3,5-dinitrosalicylate (Smith, *et al.*, 2005) and hydrogen maleate (Balasubramanian *et al.*, 1996) have been recently reported.

We report here the molecular structure of a novel compound (I) formed from the reaction of cytosine with maleic acid, namely cytosine cytosinium hydrogen maleate. It was prepared in order to extend our study on D—H···A hydrogen bonding in organic systems.

The asymmetric unit in (I) contains a hydrogen maleate anion, a cytosinium cation and a cytosine molecule which are held together by N—H···O and N—H···N hydrogen bonds (Fig. 1; Table 1). As observed in other hydrogen maleate anion, the H atom is roughly in between O1 and O3 (Madsen & Larsen, 1998).

In the crystal packing (Fig.2), cytosine bases and cytosinium cations are linked by N8A–H1N···O7A and N8B–H3N···O7B hydrogen-bonds forming a C(6)*R*^2^_2_(8) graph-set motif and yielding infinite chains running parallel to the *b* axis. These chains are connected through N–H···O and C–H···O hydrogen bonds involving the O2 and O4 atoms of the maleate thus generating R^2^_3_(10) and R^2^_2_(7) graph-set motifs (Bernstein *et al.*, 1995) and giving rise to a planar two-dimensionnal network parallel to the (1 0 1) plane (Table 1, Fig. 2).

### Experimental

The title compound was prepared by the reaction between cytosine and maleic acid. A colorless prismatic single-cristals were grown after few days of evaporation at room temperature.

### Refinement

All H atoms attached to C and N atoms were fixed geometrically and treated as riding with C—H = 0.93 Å and N—H = 0.86 Å with U_iso_(H) = 1.2U_eq_(C or N). H atom attached to O atom have been freely refined of water molecule were with U_iso_(H) = 1.5U_eq_(O).

### Crystal data

**Table:**

C_4_H_6_N_3_O^+^·C_4_H_3_O_4_^−^·C_4_H_5_N_3_O	*F*(000) = 1408
*M**_r_* = 338.29	*D*_x_ = 1.526 Mg m^−^^3^
Monoclinic, *C*2/*c*	Mo *K*α radiation, λ = 0.71073 Å
*a* = 27.3226 (5) Å	Cell parameters from 3490 reflections
*b* = 7.3618 (2) Å	θ = 2.8–28.0°
*c* = 14.6742 (4) Å	µ = 0.13 mm^−^^1^
β = 93.905 (1)°	*T* = 298 K
*V* = 2944.77 (13) Å^3^	Prism, colourless
*Z* = 8	0.3 × 0.15 × 0.1 mm

### Data collection

**Table:**

Nonius KappaCCD diffractometer	2603 reflections with *I* > 2σ(*I*)
Radiation source: fine-focus sealed tube	*R*_int_ = 0.043
graphite	θ_max_ = 28.0°, θ_min_ = 2.8°
ω–θ scans	*h* = 0→35
3490 measured reflections	*k* = 0→9
3485 independent reflections	*l* = −19→19

### Refinement

**Table:**

Refinement on *F*^2^	Primary atom site location: structure-invariant direct methods
Least-squares matrix: full	Secondary atom site location: difference Fourier map
*R*[*F*^2^ > 2σ(*F*^2^)] = 0.048	Hydrogen site location: inferred from neighbouring sites
*wR*(*F*^2^) = 0.136	H atoms treated by a mixture of independent and constrained refinement
*S* = 1.07	*w* = 1/[σ^2^(*F*_o_^2^) + (0.0596*P*)^2^ + 1.9669*P*] where *P* = (*F*_o_^2^ + 2*F*_c_^2^)/3
3485 reflections	(Δ/σ)_max_ < 0.001
202 parameters	Δρ_max_ = 0.36 e Å^−^^3^
0 restraints	Δρ_min_ = −0.23 e Å^−^^3^

### Special details

**Table:**

Geometry. All esds (except the esd in the dihedral angle between two l.s. planes) are estimated using the full covariance matrix. The cell esds are taken into account individually in the estimation of esds in distances, angles and torsion angles; correlations between esds in cell parameters are only used when they are defined by crystal symmetry. An approximate (isotropic) treatment of cell esds is used for estimating esds involving l.s. planes.
Refinement. Refinement of *F*^2^ against ALL reflections. The weighted *R*-factor *wR* and goodness of fit *S* are based on *F*^2^, conventional *R*-factors *R* are based on *F*, with *F* set to zero for negative *F*^2^. The threshold expression of *F*^2^ > σ(*F*^2^) is used only for calculating *R*-factors(gt) *etc*. and is not relevant to the choice of reflections for refinement. *R*-factors based on *F*^2^ are statistically about twice as large as those based on *F*, and *R*- factors based on ALL data will be even larger.

### Fractional atomic coordinates and isotropic or equivalent isotropic displacement parameters (Å^2^)

**Table:**

	*x*	*y*	*z*	*U*_iso_*/*U*_eq_	
O7B	0.33929 (4)	1.06422 (15)	0.29275 (9)	0.0427 (3)	
N1B	0.40074 (5)	0.90020 (18)	0.23748 (9)	0.0387 (3)	
H1B	0.4147	0.9976	0.2199	0.046*	
N3B	0.33603 (5)	0.75680 (17)	0.30317 (10)	0.0356 (3)	
H3B	0.3085	0.7616	0.3281	0.043*	
N8B	0.33306 (6)	0.44790 (19)	0.31458 (11)	0.0497 (4)	
H8B1	0.3055	0.4591	0.3390	0.060*	
H8B2	0.3452	0.3417	0.3067	0.060*	
C2B	0.35779 (6)	0.9147 (2)	0.27857 (11)	0.0345 (3)	
C4B	0.35667 (6)	0.5930 (2)	0.28930 (11)	0.0377 (4)	
C5B	0.40201 (6)	0.5833 (2)	0.24822 (12)	0.0428 (4)	
H5B	0.4171	0.4722	0.2390	0.051*	
C6B	0.42225 (6)	0.7386 (2)	0.22325 (12)	0.0429 (4)	
H6B	0.4518	0.7356	0.1954	0.052*	
O7A	0.23768 (4)	0.47389 (15)	0.38025 (9)	0.0467 (3)	
N1A	0.17582 (5)	0.63603 (19)	0.43517 (10)	0.0406 (3)	
H1A	0.1605	0.5378	0.4474	0.049*	
N3A	0.24320 (5)	0.78198 (17)	0.37790 (10)	0.0366 (3)	
N8A	0.24807 (6)	1.09115 (19)	0.37624 (12)	0.0508 (4)	
H8A1	0.2757	1.0810	0.3519	0.061*	
H8A2	0.2365	1.1970	0.3873	0.061*	
C2A	0.21960 (6)	0.6237 (2)	0.39693 (11)	0.0355 (3)	
C4A	0.22347 (6)	0.9448 (2)	0.39665 (12)	0.0378 (4)	
C5A	0.17782 (6)	0.9546 (2)	0.43695 (13)	0.0429 (4)	
H5A	0.1640	1.0659	0.4506	0.051*	
C6A	0.15542 (6)	0.7983 (2)	0.45467 (13)	0.0433 (4)	
H6A	0.1254	0.8008	0.4808	0.052*	
O1	0.00023 (4)	0.51357 (17)	0.62970 (9)	0.0448 (3)	
O2	−0.05080 (5)	0.30095 (18)	0.67256 (10)	0.0541 (4)	
O3	0.07419 (4)	0.53110 (16)	0.54870 (8)	0.0419 (3)	
H3	0.0374 (7)	0.532 (3)	0.5856 (13)	0.063*	
O4	0.12212 (5)	0.33885 (18)	0.48081 (9)	0.054	
C1	0.08607 (6)	0.3706 (2)	0.52447 (11)	0.038	
C2	0.05603 (7)	0.2114 (2)	0.54876 (14)	0.049	
H1	0.0676	0.1001	0.5293	0.059*	
C3	0.01551 (7)	0.2018 (2)	0.59356 (14)	0.0504 (5)	
H2	0.0033	0.0850	0.6003	0.061*	
C4	−0.01358 (6)	0.3478 (2)	0.63484 (12)	0.0402 (4)	

### Atomic displacement parameters (Å^2^)

**Table:**

	*U*^11^	*U*^22^	*U*^33^	*U*^12^	*U*^13^	*U*^23^
O7B	0.0443 (6)	0.0235 (6)	0.0615 (8)	0.0016 (4)	0.0113 (5)	−0.0015 (5)
N1B	0.0405 (7)	0.0322 (7)	0.0444 (8)	−0.0021 (5)	0.0104 (6)	−0.0013 (6)
N3B	0.0351 (7)	0.0245 (6)	0.0479 (8)	0.0008 (5)	0.0077 (6)	−0.0014 (5)
N8B	0.0544 (9)	0.0260 (7)	0.0704 (10)	0.0031 (6)	0.0172 (8)	0.0006 (7)
C2B	0.0369 (8)	0.0278 (8)	0.0385 (8)	0.0007 (6)	0.0013 (6)	−0.0017 (6)
C4B	0.0442 (9)	0.0267 (8)	0.0419 (9)	0.0022 (6)	0.0008 (7)	−0.0022 (6)
C5B	0.0440 (9)	0.0352 (9)	0.0500 (10)	0.0104 (7)	0.0084 (8)	−0.0028 (7)
C6B	0.0408 (9)	0.0430 (10)	0.0461 (9)	0.0061 (7)	0.0104 (7)	−0.0036 (7)
O7A	0.0452 (7)	0.0239 (6)	0.0724 (8)	0.0005 (5)	0.0135 (6)	−0.0010 (6)
N1A	0.0380 (7)	0.0313 (7)	0.0533 (8)	−0.0045 (6)	0.0099 (6)	−0.0021 (6)
N3A	0.0374 (7)	0.0215 (6)	0.0514 (8)	0.0010 (5)	0.0061 (6)	0.0001 (6)
N8A	0.0503 (8)	0.0243 (7)	0.0793 (11)	0.0016 (6)	0.0165 (8)	−0.0002 (7)
C2A	0.0361 (8)	0.0259 (8)	0.0446 (9)	0.0003 (6)	0.0029 (7)	−0.0001 (6)
C4A	0.0392 (8)	0.0277 (8)	0.0465 (9)	0.0027 (6)	0.0016 (7)	−0.0014 (7)
C5A	0.0415 (9)	0.0325 (8)	0.0550 (10)	0.0077 (7)	0.0059 (7)	−0.0060 (7)
C6A	0.0362 (8)	0.0433 (10)	0.0510 (10)	0.0031 (7)	0.0082 (7)	−0.0062 (8)
O1	0.0403 (6)	0.0392 (7)	0.0566 (7)	−0.0014 (5)	0.0154 (5)	−0.0037 (6)
O2	0.0455 (7)	0.0501 (8)	0.0694 (9)	−0.0055 (6)	0.0239 (6)	0.0023 (7)
O3	0.0418 (6)	0.0349 (6)	0.0506 (7)	−0.0033 (5)	0.0137 (5)	−0.0028 (5)
O4	0.052	0.046	0.067	0.000	0.030	−0.006
C1	0.037	0.038	0.040	−0.001	0.007	0.000
C2	0.053	0.031	0.067	0.002	0.020	−0.002
C3	0.0526 (10)	0.0309 (9)	0.0696 (12)	−0.0038 (7)	0.0179 (9)	0.0020 (8)
C4	0.0367 (8)	0.0401 (9)	0.0443 (9)	−0.0023 (7)	0.0067 (7)	0.0016 (7)

### Geometric parameters (Å, °)

**Table:**

O7B—C2B	1.2348 (19)	N3A—C2A	1.3697 (19)
N1B—C6B	1.350 (2)	N8A—C4A	1.315 (2)
N1B—C2B	1.360 (2)	N8A—H8A1	0.8600
N1B—H1B	0.8600	N8A—H8A2	0.8600
N3B—C4B	1.353 (2)	C4A—C5A	1.418 (2)
N3B—C2B	1.365 (2)	C5A—C6A	1.337 (2)
N3B—H3B	0.8600	C5A—H5A	0.9300
N8B—C4B	1.314 (2)	C6A—H6A	0.9300
N8B—H8B1	0.8600	O1—C4	1.281 (2)
N8B—H8B2	0.8600	O1—H3	1.25 (2)
C4B—C5B	1.416 (2)	O2—C4	1.239 (2)
C5B—C6B	1.332 (2)	O3—C1	1.282 (2)
C5B—H5B	0.9300	O3—H3	1.17 (2)
C6B—H6B	0.9300	O4—C1	1.2333 (19)
O7A—C2A	1.2398 (19)	C1—C2	1.488 (2)
N1A—C6A	1.357 (2)	C2—C3	1.327 (3)
N1A—C2A	1.358 (2)	C2—H1	0.9300
N1A—H1A	0.8600	C3—C4	1.490 (3)
N3A—C4A	1.3503 (19)	C3—H2	0.9300
			
C6B—N1B—C2B	122.40 (14)	H8A1—N8A—H8A2	120.0
C6B—N1B—H1B	118.8	O7A—C2A—N1A	121.00 (14)
C2B—N1B—H1B	118.8	O7A—C2A—N3A	121.13 (14)
C4B—N3B—C2B	121.71 (13)	N1A—C2A—N3A	117.87 (13)
C4B—N3B—H3B	119.1	N8A—C4A—N3A	117.61 (15)
C2B—N3B—H3B	119.1	N8A—C4A—C5A	122.06 (15)
C4B—N8B—H8B1	120.0	N3A—C4A—C5A	120.33 (15)
C4B—N8B—H8B2	120.0	C6A—C5A—C4A	117.66 (15)
H8B1—N8B—H8B2	120.0	C6A—C5A—H5A	121.2
O7B—C2B—N1B	121.34 (14)	C4A—C5A—H5A	121.2
O7B—C2B—N3B	121.59 (14)	C5A—C6A—N1A	121.10 (15)
N1B—C2B—N3B	117.07 (13)	C5A—C6A—H6A	119.4
N8B—C4B—N3B	117.68 (15)	N1A—C6A—H6A	119.4
N8B—C4B—C5B	122.64 (15)	C4—O1—H3	112.6 (11)
N3B—C4B—C5B	119.67 (15)	C1—O3—H3	111.8 (12)
C6B—C5B—C4B	117.72 (15)	O4—C1—O3	123.04 (15)
C6B—C5B—H5B	121.1	O4—C1—C2	116.62 (16)
C4B—C5B—H5B	121.1	O3—C1—C2	120.34 (14)
C5B—C6B—N1B	121.38 (15)	C3—C2—C1	130.78 (17)
C5B—C6B—H6B	119.3	C3—C2—H1	114.6
N1B—C6B—H6B	119.3	C1—C2—H1	114.6
C6A—N1A—C2A	122.13 (14)	C2—C3—C4	130.43 (16)
C6A—N1A—H1A	118.9	C2—C3—H2	114.8
C2A—N1A—H1A	118.9	C4—C3—H2	114.8
C4A—N3A—C2A	120.90 (13)	O2—C4—O1	123.04 (16)
C4A—N8A—H8A1	120.0	O2—C4—C3	117.21 (16)
C4A—N8A—H8A2	120.0	O1—C4—C3	119.74 (14)

### Hydrogen-bond geometry (Å, °)

**Table:**

*D*—H···*A*	*D*—H	H···*A*	*D*···*A*	*D*—H···*A*
N1A—H1A···O4	0.86	1.89	2.7426 (19)	174
N1B—H1B···O2^i^	0.86	1.91	2.7701 (19)	174
N8A—H8A1···O7B	0.86	2.00	2.8582 (19)	178
N8A—H8A2···O7A^ii^	0.86	2.04	2.8329 (19)	153
N3B—H3B···N3A	0.86	1.98	2.8370 (19)	176
N8B—H8B1···O7A	0.86	1.99	2.8458 (19)	173
N8B—H8B2···O7B^iii^	0.86	2.06	2.8491 (18)	153
O3—H3···O1	1.17 (2)	1.25 (2)	2.4167 (16)	173 (2)
C6B—H6B···O1^i^	0.93	2.50	3.186 (2)	131
C5B—H5B···O2^iv^	0.93	2.42	3.330 (2)	165
C5A—H5A···O4^ii^	0.93	2.37	3.296 (2)	175

Symmetry codes: (i) *x*+1/2, −*y*+3/2, *z*−1/2; (ii) *x*, *y*+1, *z*; (iii) *x*, *y*−1, *z*; (iv) *x*+1/2, −*y*+1/2, *z*−1/2.
